# SARS-CoV-2 viral load is associated with risk of transmission to household and community contacts

**DOI:** 10.1186/s12879-022-07663-1

**Published:** 2022-08-05

**Authors:** Darlene Bhavnani, Emily R. James, Kaitlyn E. Johnson, Sylvie Beaudenon-Huibregtse, Patrick Chang, Paul J. Rathouz, Minda Weldon, Andreas Matouschek, Amy E. Young

**Affiliations:** 1 Department of Population Health, Dell Medical School, University of Texas at Austin, Austin, USA; 2 UT Health Austin, Dell Medical School, University of Texas at Austin, Austin, USA; 3 Department of Integrative Biology, University of Texas at Austin, Austin, USA; 4 High Throughput Testing Core, University of Texas at Austin, Austin, USA; 5 Epidemiology and Disease Surveillance Unit, Austin Public Health, Austin, USA; 6 Department of Molecular Biosciences, The University of Texas at Austin, Austin, USA

**Keywords:** COVID-19, Viral load, Contact, Tracing, Transmission, Exposure

## Abstract

**Background:**

Factors that lead to successful SARS-CoV-2 transmission are still not well described. We investigated the association between a case’s viral load and the risk of transmission to contacts in the context of other exposure-related factors.

**Methods:**

Data were generated through routine testing and contact tracing at a large university. Case viral loads were obtained from cycle threshold values associated with a positive polymerase chain reaction test result from October 1, 2020 to April 15, 2021. Cases were included if they had at least one contact who tested 3–14 days after the exposure. Case-contact pairs were formed by linking index cases with contacts. Chi-square tests were used to evaluate differences in proportions of contacts testing positive. Generalized estimating equation models with a log link were used to evaluate whether viral load and other exposure-related factors were associated with a contact testing positive.

**Results:**

Median viral load among the 212 cases included in the study was 5.6 (1.8–10.4) log_10_ RNA copies per mL of saliva. Among 365 contacts, 70 (19%) tested positive following their exposure; 36 (51%) were exposed to a case that was asymptomatic or pre-symptomatic on the day of exposure. The proportion of contacts that tested positive increased monotonically with index case viral load (12%, 23% and 25% corresponding to < 5, 5–8 and > 8 log_10_ copies per mL, respectively; *X*^*2*^ = 7.18, df = 2, *p* = 0.03). Adjusting for cough, time between test and exposure, and physical contact, the risk of transmission to a close contact was significantly associated with viral load (RR = 1.27, 95% CI 1.22–1.32).

**Conclusions:**

Further research is needed to understand whether these relationships persist for newer variants. For those variants whose transmission advantage is mediated through a high viral load, public health measures could be scaled accordingly. Index cases with higher viral loads could be prioritized for contact tracing and recommendations to quarantine contacts could be made according to the likelihood of transmission based on risk factors such as viral load.

**Supplementary Information:**

The online version contains supplementary material available at 10.1186/s12879-022-07663-1.

## Background

COVID-19 is caused by severe acute respiratory syndrome coronavirus 2 (SARS-CoV-2) and has led to significant morbidity and mortality worldwide. By November 11, 2021, more than 250 million COVID-19 cases and more than 5 million associated deaths had been reported [1]. SARS-CoV-2 is transmitted from person-to-person through aerosolized particles exhaled by infectious individuals [2]. Public health control efforts have largely relied on the detection and isolation of cases and quarantine of close contacts in order to prevent further transmission. Factors that drive successful transmission are still not well understood.

Secondary attack rates appear to be highest after household [3–5] or congregate setting exposures [6] where risk of transmission may be exacerbated by prolonged and close contact, poor ventilation, and limited use of masks. Outbreaks have also been documented in settings such as fitness facilities [7], schools [8], and workplaces [9] that share some of these same factors. At the same time, transmission may be highly variable within these settings. Published estimates of secondary attack rates in the household are as low as 10% [10] and as high as 30% [11]. While some have reported that symptomatic persons are more likely to transmit SARS-CoV-2 [10, 12], symptoms that may emit droplets such as sneezing and coughing do not consistently explain this variability [4, 13]. Furthermore, asymptomatic or pre-symptomatic individuals are expected to account for at least half of all transmission events [14–16]. Emerging evidence suggests that difference in secondary attack rates may be related to differences in viral load [17–19].

Differences in viral load have been investigated in a number of studies. Some have found similar distributions of viral load in children and adult [20, 21], vaccinated and unvaccinated [22] and symptomatic and asymptomatic cases [17, 23, 24] while others have reported significantly higher viral loads in unvaccinated [25] and symptomatic cases [20, 21]. Yet, to date, there have been relatively few epidemiological studies that have addressed the role of viral load on transmission to close contacts [4, 26–29]. Some have been limited in scope focusing on symptomatic cases [4, 26] or cases tested and treated at a hospital [27], while others have restricted contacts to those in university residence halls [28] and in the household [29]. Specimens collected over time from infected individuals demonstrate that viral loads increase, peak around the time of symptom onset and gradually decline for 7–10 days [15, 24]. Relative risk of transmission to close contacts is significantly higher in the 2 days prior to and 3 days following symptom onset [10]. Given the dynamic pattern of viral shedding and infectiousness, we sought to simultaneously consider viral load, the timing of exposure relative to the case’s test and specific symptoms such as cough and runny nose. We included in our study population asymptomatic and symptomatic cases detected by a university-wide testing program, taking advantage of detailed case investigations and contact tracing that extended beyond the university setting to describe and evaluate the role of viral load on transmission.

## Methods

### Study site

Data were generated by the University of Texas at Austin’s (UT) COVID-19 testing and tracing program. While UT shifted towards remote learning and work in the fall of 2020, certain classes, student activities, research and operations remained in-person during the fall 2020 and spring 2021 semesters. Various testing programs were available on campus including a voluntary testing campaign [30]. Positive and negative test results from all campus testing programs were reported to UT Contact Tracing, which was housed within Dell Medical School and functioned under the authority of Austin Public Health.

### Study population

UT students, faculty and staff who tested positive for SARS-CoV-2 through the voluntary testing campaign at UT between October 1, 2020 and April 15, 2021 as well as their contacts were included in the study. Cases were included if they were: (1) successfully investigated at UT; (2) willing to share their contacts; and (3) had at least one contact who tested 3 to 14 days after the exposure. Cases were excluded from the study if they were hospitalized or vaccinated. Contacts were excluded from study if they had other documented exposures in the 2 weeks prior to their test, had a known previous infection, or were vaccinated. Case and contact pairs were manually excluded if the case exposed the contact at an event with at least 10 persons in attendance. In order to rule out the possibility that the contact transmitted the virus to the case, pairs were also excluded if the contact’s symptom onset occurred before the case’s onset, or if case and contact onset dates were too close together (0–2 days).

### Contact tracing

Contact tracers made up to three attempts to investigate cases by phone. Case investigations included questions about demographic characteristics, symptoms, and comorbidities or conditions. The latter included smoking, pregnancy, diabetes, cardiac disease, hypertension, chronic pulmonary disease, asthma, chronic kidney disease, chronic liver disease, or being immunocompromised. Interviews also captured exposures during the case’s infectious period, as defined in [30]. Test date was used as a proxy for symptom onset for asymptomatic cases. Exposure details including location, duration and mask use were obtained from the case. Cases were asked for the names and phone numbers of close contacts. Close contacts were defined as anyone within 6 feet of the case for 15 min or more, or anyone who made physical contact with the case during the case’s infectious period. Contact tracers made up to three attempts to notify close contacts of the exposure by phone. Recent test history was collected from contacts, including type of test. Contacts were encouraged to test immediately if experiencing any new symptoms or test 3–7 days from the exposure if no symptoms developed. Contact tracers helped to schedule tests on campus and provided information about where to access testing in the community. When possible, follow up calls were made to contacts 7–10 days from the exposure to obtain results of tests that may have occurred off campus. In collaboration with Austin Public Health, UT Contact tracing verified positive test results of contacts residing in Travis County and identified additional UT staff, faculty, and student cases who were interviewed by Austin Public Health.

### Laboratory methods

During UT’s voluntary testing, saliva samples were self-collected under the supervision of a health care provider, using the OMNIgene^®^ ORAL OM-505 collection device which stabilizes viral RNA from saliva specimens, following instructions from the manufacturer (DNAgenotekTM, INC, Canada). Samples were transferred at room temperature to the University of Texas in Austin’s High Throughput Testing Core (HTTC) CLIA laboratory (CLIA license # 45D2183984) for processing and testing for the presence of SARS-CoV-2. Testing was performed using HTTC’s laboratory-developed SARS-CoV-2 test for saliva, using ThermoFisher’s TaqPath COVID-19 Combo Kit assay, a qualitative real-time qPCR assay that was awarded Emergency-Use Authorization (EUA) for the detection of SARS-CoV-2 viral RNA in upper respiratory specimens. This assay is a multiplex assay that contains three primer/probe sets specific to three different SARS-CoV-2 regions located in the ORF1a/b, the N gene and the S gene, respectively, plus primers/probes for bacteriophage MS2 which is used as an internal process control for RNA extraction and amplification.

RNA extraction was performed from 200 uL of saliva using ThermoFisher’s Applied Biosystems™ MagMax Viral/Pathogen II Nucleic Acid Isolation Kit on either an automated Hamilton Star^®^ Biorobot (Hamilton Company, USA) or a KingFisher Flex System instrument (Thermo Scientific™, USA) and eluted in a volume of 50 uL of elution buffer. Following RNA extraction, RT-qPCR was immediately performed using the TaqPath COVID-19 Combo Kit with 10 uL of extracted RNA sample. RT-qPCR was performed on a ThermoFisher’s Applied Biosystems™ QuantStudio 7 Flex Real-Time PCR system equipped with ExpressionSuite software v1.3. Reports were generated using ThermoFisher’s Applied Biosystems™ COVID-19 Interpretive Software v2.5. Each viral gene target had a cut-off cycle threshold (Ct) of < 37, and the presence of at least two primer/probe sets were used by the Interpretive Software to report a positive result, as determined by the manufacturer (ThermoFisher). Viral load was measured using standardization curves developed from Ct values (Additional file 1: Methods).

### Statistical analysis

Ct values of the three primer/probe sets were compared before producing estimates of viral load (expressed as log_10_ RNA copies per mL of saliva, henceforth viral load). Using t-tests, the distribution of viral load was compared between cases that reported specific symptoms, such as cough, on the day of exposure, as well as between cases with and without a comorbidity. Cases were grouped by viral load (< 5, 6–8, > 8) and separately, by symptom presentation on the day of exposure. The proportion of contacts that tested positive following exposure to an infectious case were compared across groups using Chi-square tests. Given that viral load changes throughout the course of infection, a sensitivity analysis was performed by restricting the sample to cases that tested on the day of exposure to their contacts.

Univariable and multivariable Generalized Estimating Equations models with a log link were used to estimate the effects of potential risk factors on transmission among case-contact pairs. The outcome of interest was the contact’s test result (positive or negative). The model form accounted for the extra binomial variation arising from index cases who had multiple contacts [31]. Potential risk factors included viral load at the time of test, number of days between test and exposure, the presentation of any symptom on the day of exposure, cough and congestion or runny nose on the day of exposure, mask use by case and contact during the exposure, contact type (physical contact versus non-physical and within 6 feet or in a shared indoor space), duration of contact (< 30 min or ≥ 30 min) and household contact. Exposures on the day of symptom onset and post-symptom onset were categorized together. Associations were expressed in terms of risk ratios (RR) and 95% confidence intervals (95% CI) were used to evaluate statistical significance. Data analysis and model execution were performed in Python and R. This study was determined to be non-human subjects research by the University of Texas Institutional Review Board.

## Results

From October 1, 2020 to April 15, 2021, 1016 cases tested positive for SARS-CoV-2 through UT’s voluntary testing program. Four cases were hospitalized and excluded from the study. All cases were attempted to be investigated; 895 (88%) were successfully investigated and of those, 656 cases reported contacts. There were 287 index cases with at least one contact who: (1) tested 3–14 days following the exposure; (2) had no other known exposures to COVID-19 in the 14 days prior to their test; and (3) had no known history of SARS-CoV-2 infection. Twenty-five cases were excluded because either case or contact in a pair had received at least one dose of a COVID-19 vaccine by the time of exposure. Following manual inspection of case-contact pairs, 50 cases were excluded due to uncertainty about the direction of transmission, resulting in 212 index cases for analysis.

**Fig. 1:**
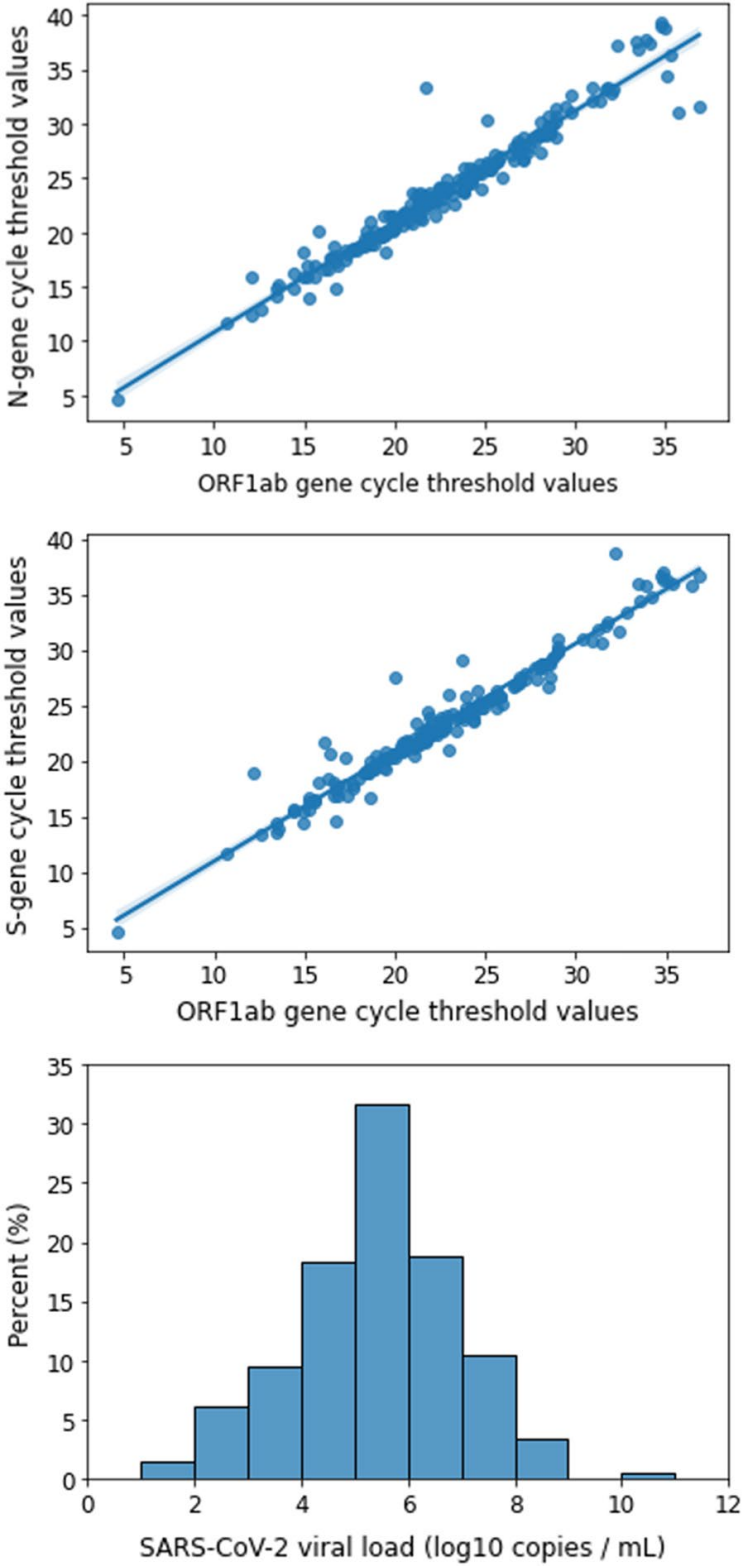
Correlation between N-gene cycle threshold (Ct) values and ORF1ab gene Ct values among index cases (top). Correlation between S-gene cycle threshold (Ct) values and ORF1ab gene Ct values among index cases (middle). Percent of index cases by SARS-CoV-2 viral load in saliva, estimated using ORF1ab Ct values (bottom)

Index cases were predominantly students with a median age of 21 years (Table 1). Approximately, 56% of cases were female, 14% had a comorbidity, and 79% had presented with symptoms by the time of investigation. Among cases with symptoms, 64% reported congestion or runny nose, 43% reported cough and 89% tested on or after the day of symptom onset. Cycle threshold values of the three genes used to detect SARS-CoV-2 were highly correlated (Fig. 1). Viral loads were estimated using the ORF1ab gene given that it was consistently detected among all test-positives and less likely to vary among variants containing mutations on the spike protein. The median viral load among index cases was 5.6 log_10_ copies of RNA per mL of saliva (range = 1.8–10.4). A small proportion of cases (4%) had viral loads > 8 log_10_ RNA copies per mL. Only one case (< 1%) had a viral load > 9 log_10_ copies per mL. Cases reporting one or more comorbidities also had higher viral loads compared to those with no comorbidities (5.83 log_10_ copies per mL vs. 5.31 log_10_ copies per mL, *t* = 2.05, *p* = 0.04, Additional file 1: Figure S1). Cases presenting with cough on the day of exposure had significantly higher viral loads compared to cases with no cough (5.79 log_10_ copies per mL vs. 5.21 log_10_ copies per mL respectively, *t* = 2.5, *p* = 0.01, Additional file 1: Figure S2). No significant differences in viral load were found between cases with and without congestion or runny nose (5.59 log_10_ copies per mL vs. 5.42 log_10_ copies per mL, *t* = 2.03, *p* = 0.05, Additional file 1: Figure S3).

**Table 1:** Characteristics of 212 index cases diagnosed at the University of Texas at Austin, October 1, 2020–April 15, 2021

	N (%) or median (range)
Age, years	21 (18–58)
Sex	
Female	117 (55.5%)
Viral load (log10 copies per mL)	5.6 (1.8–10.4)
< 5 log10 copies per mL	75 (35.4%)
5–8 log10 copies per mL	129 (60.8%)
> 8 log10 copies per mL	8 (3.8%)
Comorbidity	
Yes	29 (13.7%)
Asthma	21 (72.4%)
Current smoker	3 (10.3%)
Chronic kidney disease	2 (6.9%)
Diabetes	1 (3.4%)
Severe obesity (BMI > 35)	1 (3.4%)
Immunocompromised	1 (3.4%)
Hypertension	1 (3.4%)
Symptoms reported at time of investigation	
Yes	167 (78.8%)
Congestion or runny nose*	107 (64.1%)
Fatigue*	72 (43.1%)
Cough*	72 (43.1%)
Headache*	72 (43.1%)
Sore throat*	68 (40.7%)
Muscle ache*	40 (24.0%)
Loss of taste or smell*	36 (21.6%)
Test relative to symptom onset (N = 167)	
Tested prior to symptom onset	19 (11.4%)
Tested on day of onset	43 (25.7%)
Tested after symptom onset	105 (62.9%)
University affiliation	
Student	200 (94.4%)
Staff or Faculty	12 (5.6%)

^*^Symptom reported by at least 20% of symptomatic cases (N = 167)

**Fig. 2:**
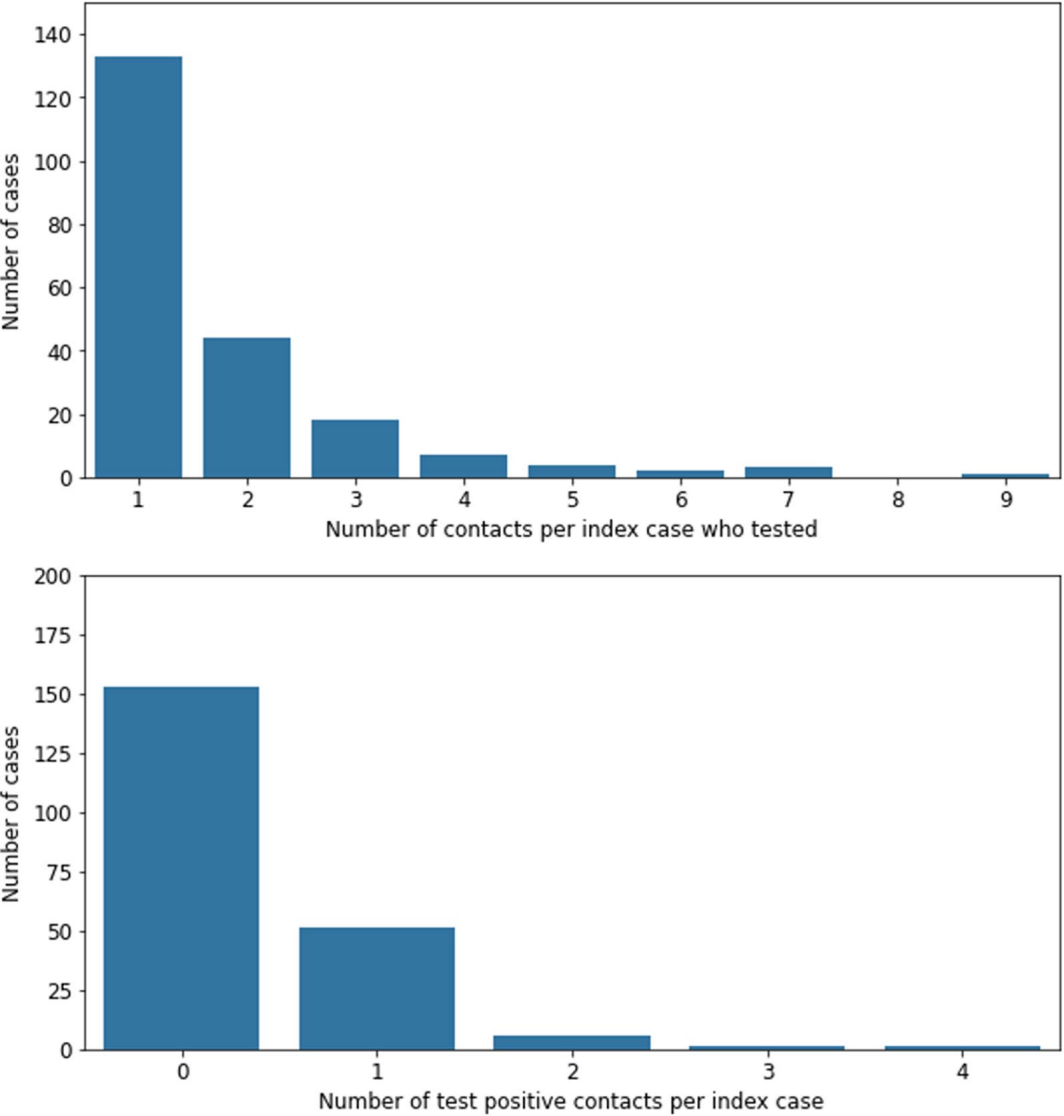
The number of index cases detected between October 1, 2020 and April 15, 2021 at UT Austin by the number of close contacts that tested (top) and number of test-positive contacts (bottom)

There were 365 close contacts that met inclusion criteria. Most cases (62%) were associated with only one close contact who met the inclusion criteria (Fig. 2). Contacts ranged in age from 16 to 92 years (median = 21 years) and were predominantly female (54%); 15% reported comorbidities and 89% were students, staff or faculty at UT.

Just over half of all contacts were exposed to an asymptomatic or pre-symptomatic case (Table 2). The majority of exposures were indoors (90%), lasted greater than 30 min (90%), involved no mask use by the case and contact (83%) and non-physical contact (87%). Approximately 41% of contacts were household contacts.

**Table 2:** Attributes of exposures between index cases and their close contacts (N = 365)

Exposure attribute	N (%)
Symptomatic status of index case on day of exposure (N = 365)	
Asymptomatic	76 (20.8)
Pre-symptomatic	115 (31.5)
Symptom onset	51 (14.0)
Post symptom onset	123 (33.7)
Case coughing at exposure	
Yes	57 (15.6)
No	308 (84.4)
Case had congestion or runny nose at exposure	
Yes	115 (31.5)
No	250 (68.5)
Viral load of index case at time of test, log_10_ copies per mL (N = 365)	
< 5	134 (36.7)
5–8	219 (60.0)
> 8	12 (3.3)
Location (N = 365)	
Indoor	330 (90.4)
Outdoor	35 (9.6)
Duration (N = 349)	
< 30 min	35 (10.0)
≥ 30 min	314 (90.0)
Mask use (N = 363)	
No masks used	300 (82.6)
Case and/or contact wore a mask	63 (17.4)
Contact type (N = 362)	
Physical contact	48 (13.3)
Non-physical contact	314 (86.7)
Contact relationship (N = 365)	
Household	149 (40.8)
Non-household	216 (59.2)

**Fig. 3:**
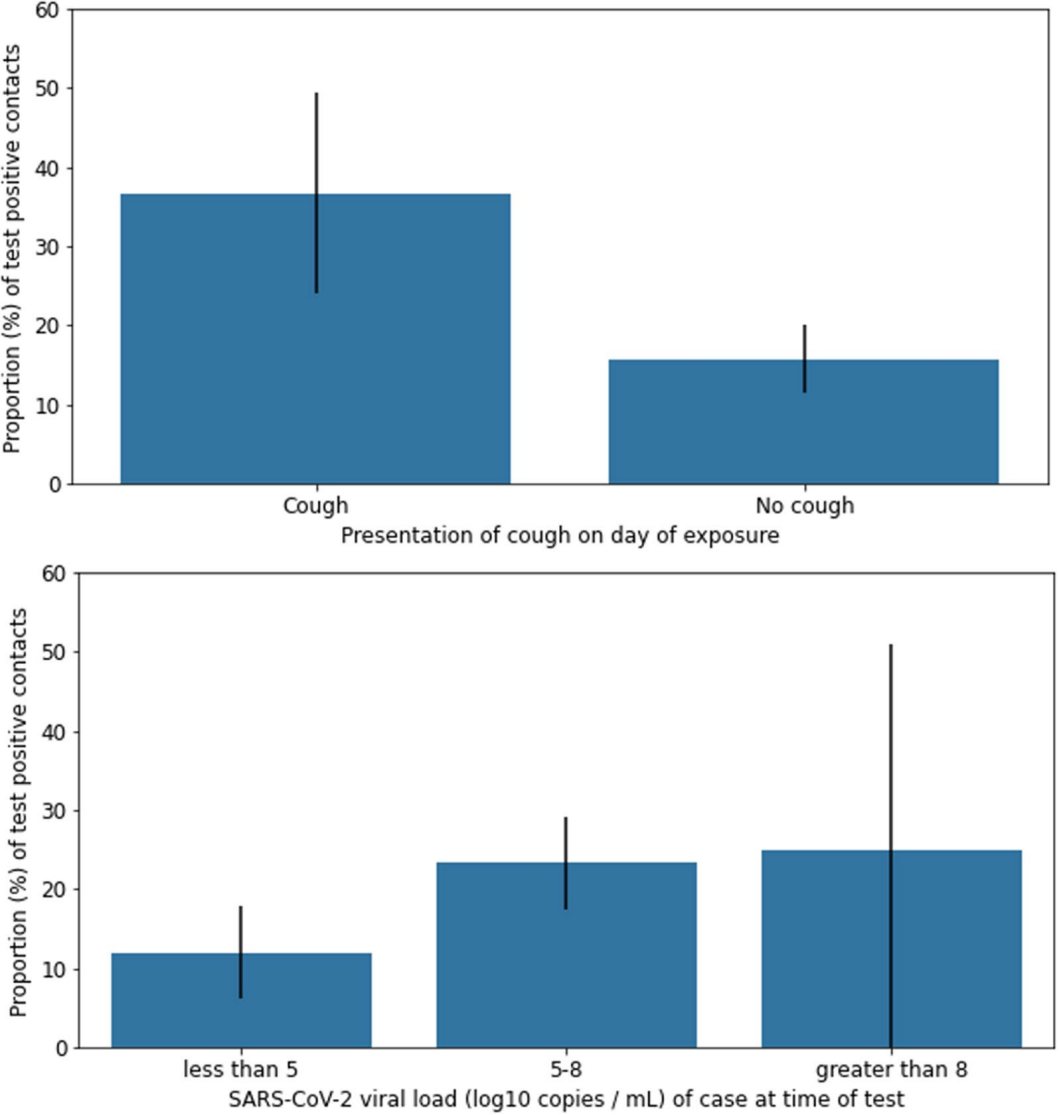
Proportion of test positive contacts and 95% confidence intervals by presentation of cough among index cases on the day of exposure (top) and viral load at the time of test (bottom)

Out of 365 contacts, 343 (94%) tested for SARS-CoV-2 using a nucleic acid amplification test, 12 (3%) tested using an antigen test and 10 (3%) had an unknown test type but were able to provide the date and location of their test. Among all contacts that tested, 70 (19%) tested positive for the virus; 36 (51%) were exposed to an asymptomatic or pre-symptomatic case and 54 (77%) were exposed to a case with viral load ≥ 5 log10 copies per mL. The proportion of contacts that tested positive increased monotonically with viral load of the index case at the time of test (12%, 23% and 25% corresponding to < 5, 5–8 and > 8 log_10_ copies per mL, respectively; *X*^2^ = 7.18, df = 2, *p* = 0.03; Fig. 3). The proportion of contacts that tested positive was highest after exposure to cases with cough compared to cases with no report of cough (35% vs. 16%, respectively; *X*^2^ = 9.85, df = 1, *p* = 0.002). The difference in test positivity of contacts by congestion or runny nose of index case was not statistically significant (Additional file 1: Table S1). When exposures were restricted to those outside the home (N = 190), test positivity significantly increased with viral load (11%, 27% and 33% corresponding to < 5, 5–8 and > 8 log_10_ copies per mL, *X*^2^ = 7.04, df = 2, *p* = 0.03, Additional file 1: Table S2). In this restricted sample, test positivity was higher among those exposed to cases with cough compared to cases without cough (44% vs 19%, df = 1, *X*^2^ = 5.08, *p* = 0.02). The majority of exposures occurred within 2 days of the case’s test (Additional file 1: Figure S4). When exposures were restricted to those on the day of the index case’s test (N = 75), test positivity was higher among contacts exposed to cases with viral loads 5–8 log_10_ copies per mL compared to contacts exposed to cases with viral loads < 5 log_10_ copies per mL (28% vs. 3%, respectively) and among contacts exposed to cases with cough compared to cases without cough (43% vs 8%, df = 1, *X*^2^ = 8.34, *p* = 0.004).

The risk of transmission to a close contact was significantly associated with viral load (adjusted RR = 1.27 per log_10_ increase in viral load, 95% CI 1.22–1.32, Table 3). Risk of transmission was also associated with the number of days between test and exposure (adjusted RR = 1.14, 95% CI 1.11–1.17), the index case presenting with cough on the day of exposure (adjusted RR = 1.41, 95% CI 1.04–1.92) and physical contact (adjusted RR = 1.78, 95% CI 1.26–2.53). These associations remained statistically significant when viral load was categorized as < 5, 5–8 and > 8 log_10_ copies per mL (Additional file 1: Table S3). Little to no correlation was found between risk factors in the final model (Variance Inflation Factor range = 1.1–1.8). Factors not associated with transmission in this population included age, presentation of any symptom, mask use, duration of exposure, location of exposure and household contact.

**Table 3:** Association between exposure attributes and a contact’s positive test result

Characteristic	Unadjusted RR (95% CI)	*p*-value	Adjusted RR* (95% CI)	*p*-value
Index case viral load at time of test (log_10_ RNA copies per mL of saliva)	1.25 (1.06–1.46)	0.007	1.27 (1.22–1.32)	*p* < *0.001*
Time between index case test and exposure (days)	1.12 (1.10–1.15)	*p* < 0.001	1.14 (1.11–1.17)	*p* < 0.001
Age of case (years)	0.99 (0.94–1.04)	0.7	–	–
Case comorbidities	0.89 (0.45–1.74)	0.7	–	–
Symptomatic status on day of exposure				
Asymptomatic	Reference	–	–	–
Pre-symptomatic	2.23 (1.00–4.97)	0.05	–	–
Symptomatic	2.05 (0.94–4.48)	0.07	–	–
Case had cough on day of exposure	1.85 (1.15–2.99)	0.01	1.41 (1.04–1.92)	0.03
Case congested or with runny nose on day of exposure	1.46 (0.94–2.29)	0.09	–	–
Mask use				
No masks used	Reference	–	–	–
Case and/or contact wore a mask	1.02 (0.59–1.78)	0.9	–	–
Duration (min)				
< 30	Reference	–	–	–
≥ 30	1.61 (0.84–3.09)	0.1	–	–
Location				
Outdoor	Reference	–	–	–
Indoor	2.39 (0.69–8.24)	0.2	–	–
Contact type				
Non-physical contact	Reference	–	–	–
Physical contact	2.14 (1.34–3.44)	0.001	1.78 (1.26–2.53)	0.001
Contact relationship				
Household	Reference	–	–	–
Non-household	1.19 (0.77–1.83)	0.4	–	–

^*^Adjusted for viral load, time between index case test and exposure with contact, cough and contact type

## Discussion

Viral load of the index case, cough, and having a physical interaction with a contact appear to be the most important factors associated with SARS-CoV-2 transmission among unvaccinated case-contact pairs. We found that 77% of transmission events were associated with case viral loads of ≥ 1 × 10^5^ copies of viral RNA per mL of saliva. Our findings are consistent with others [4, 26–29] and yet, they account for the timing of exposure relative to the measurement of viral load. Importantly, approximately 70% of index cases included in our study did not appear to transmit SARS-CoV-2. Extremely high viral loads (≥ 1 × 10^9^ copies per mL) were found in just one case. Similar results were reported from a testing program at the University of Colorado during the fall of 2020. Using an interpolated probability density function, investigators summed the viral load across cases starting with those with the highest viral load. They found that just 2% of individuals harbored 90% of the total viral load [17]. These results are consistent with the notion that SARS-CoV-2 is propagated through superspreading events [32].

Our findings have important implications for the test-trace-isolate strategy. Rapid antigen tests, which can provide results within minutes, may be a better marker of infectiousness than other more sensitive tests, such as PCR. One study of four rapid antigen test kits reported that their ability to detect infection was > 90% when viral loads were ≥ 1 × 10^5^ RNA copies per mL [33]. Increased reliance on rapid antigen testing could help to isolate infectious cases sooner. When nucleic acid amplification (e.g. PCR) tests are used, viral loads could help to prioritize contact tracing. The effectiveness of contact tracing is highly dependent on rapid quarantine and quarantine of a large proportion of contacts [34, 35]. During epidemic peaks, public health agencies that rely on manual methods to notify contacts are often too limited in resources to keep pace [36]. In such instances, notifying contacts of the most infectious cases, such as those with higher viral loads, could increase the impact of the strategy on transmission during a surge.

While our study period predates several subsequent surges attributed to newer variants of concern, data presented on viral load and transmission may help to contextualize the high transmissibility of variants such as Alpha and Delta. Higher transmissibility of these variants compared to the wildtype have been hypothesized to be linked to more efficient cell surface entry [37] and a suppression of the interferon response within the cell, allowing the virus to replicate to higher levels [38]. However, with lower infectious viral loads compared to Delta, Omicron’s transmission advantage may be mediated by factors other than viral load [39]. More research is needed to better understand the mechanisms of infection, including whether higher viral loads lead to increased concentrations of virus emitted through droplets and or increased aerosolization of the virus [40].

Whether symptoms such as cough are simply a consequence of higher viral loads, or help to perpetuate transmission independent of viral load, is critical to address. Our results demonstrate that viral load at the time of test is associated with cough on the day of exposure. And, cough on the day of exposure is associated with increased risk of transmission. These results are only partially consistent with those reported from 282 case-contact pairs in Spain where cough was significantly associated with higher viral load but was not associated with increased risk of transmission [4]. Furthermore, kinetic models of transmission demonstrate that talking and singing can shed viral loads comparable to coughing through large droplets and aerosols [41]. While cough was found to be an important risk factor, we found that exposures to an asymptomatic or pre-symptomatic case accounted for just over half of transmission events. The substantial proportion of transmission from persons without symptoms, underscores the importance of routine use of low barrier testing.

There are a number of study limitations that should be noted. First, we restricted our study population to cases with at least one contact that tested following the exposure. Contacts that tested may be more likely to have been exposed to someone with symptoms (correlated with viral load [20, 21]). And, contacts that tested may be more likely to have symptoms like cough themselves (correlated with test positivity), introducing some selection bias. Nonetheless, when we looked at all cases investigated during the study timeframe, we found no association between their symptom presentation and their contact having tested, demonstrating the low likelihood of this bias. Second, by excluding case-contact pairs with symptom onset dates within 2 days of each other, we may have removed cases that transmitted to contacts at or near to their peak viral load. If we had excluded cases with high viral loads, this would have led to more conservative estimates of effect. Third, we used viral load at the time of the test as a proxy for viral load at the time of exposure. Given that viral load is dynamic throughout the course of infection, this assumption could have led to some misrepresentation of viral load at the time of exposure. And yet, when our analysis was limited to exposures on the day of a test, our conclusions did not change. Fourth, our analysis of exposure is based on the last date of contact which is less applicable to household contacts who may be exposed over multiple days. This could have resulted in the classification of an exposure to a single case that was pre-symptomatic and then became symptomatic, as an exposure to a symptomatic case. However, a sensitivity analysis restricted to non-household contacts with single-day exposures, resulted in similar findings. Fifth, the skewed distribution of some protective factors, such as masks, in our study population may have restricted us from finding statistically significant associations with transmission. Sixth, we used date of symptom onset and specific symptoms collected by case investigators to estimate whether the case presented with symptoms, like cough, on the day of exposure. If the presentation of symptoms was inconsistent following onset, cough may have been misclassified. Finally, viral load dynamics and transmission potential has been shown to differ by age [26, 29]. Given, the narrow age distribution of our study population, the generalizability of our findings to other populations may be limited.

Our study has the advantage that it included the use of consistent targets, primers and probes among all test-positives, limiting inter-assay variability in cycle threshold values. Furthermore, we interrogated the impact of viral load along with other exposure details including the time between measurement of viral load and exposure. It would be important to understand whether these relationships persist for newer variants. And, where they do, the public health community could take better advantage of viral load generated through nucleic acid amplification tests. Viral load could be used to prioritize cases for contact tracing as well as to scale quarantine and testing measures for close contacts, which some have suggested [42] could improve compliance.

## Supplementary Information

**Additional file 1. **Supplemental Data and Methods.

## Data Availability

All data generated or analyzed during this study are included in this published article and its additional information files.
