# Supplementary material for: SARS-CoV-2 viral load is associated with risk of transmission to household and community contacts
Source: BMC Infect Dis. 2022 Aug 5;22:672. doi: 10.1186/s12879-022-07663-1 (PMC9354300; doi:10.1186/s12879-022-07663-1)
Supplement: Supplementary file 1 — Additional file 1. Supplemental Data and Methods. [file 12879_2022_7663_MOESM1_ESM.docx]

**Supplemental Data**

**Figure S1. Distribution of SARS-CoV-2 viral load by presence or absence of**  **comorbidities**


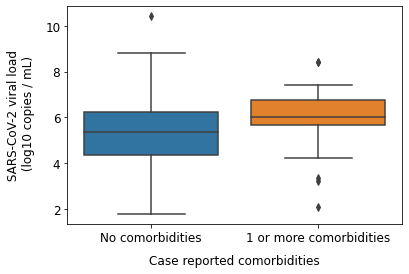


**Figure S2. Distribution of SARS-CoV-2 viral load by presentation of cough on day of exposure**


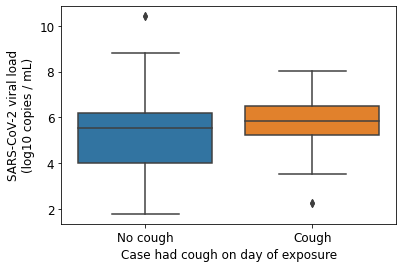


**Figure S3. Distribution of SARS-CoV-2 viral load by congestion or runny nose on day of exposure**


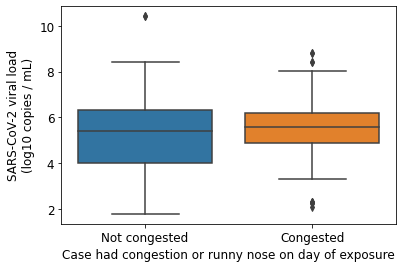


**Figure S4. Distribution of days between index case’s test and exposure**


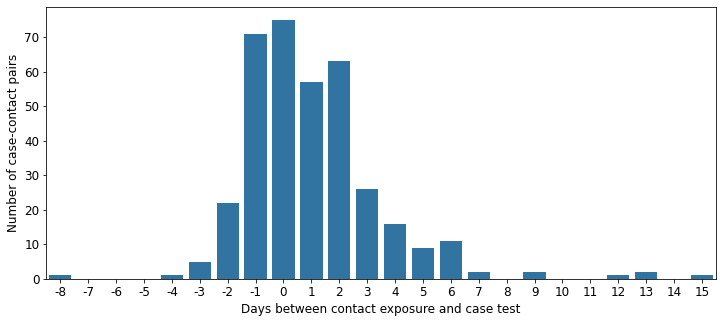


The distribution between the index case’s positive test and exposure between the case and their contact ranged from -8 to 15 days. Negative values indicate that the index case tested before the exposure. Positive values indicate that the index case tested after the exposure.

**Table S1. Proportion of test-positive contacts by presentation of congestion or runny nose among index cases on the day of exposure**

|  | Number of contacts | Test-positive contacts (%) |
| --- | --- | --- |
| Case had congestion or runny nose | 115 | 28 (24.3) |
| No congestion or runny nose | 250 | 42 (16.8) |

*X^2^*= 2.4, df = 1, *p* = 0.1

**Table S2. Sensitivity analysis of the proportion of test-positive contacts by viral load and symptomatic status.**

| Sensitivity Analyses | Number of contacts | Test positive contacts (%) |
| --- | --- | --- |
| Non-household contacts with single day exposures (N=190) |  |  |
| Viral load of index case at time of test, log_10_ copies per mL |  |  |
| < 5 | 72 | 8 (11.1) |
| 5-8 | 112 | 30 (26.8) |
| >8 | 6 | 2 (33.3) |
| Case presented with cough on day of exposure |  |  |
| Yes | 18 | 8 (44.4) |
| No | 172 | 32 (18.6) |
| Exposures on day of index case’s test (N=75) |  |  |
| Viral load of index case at time of test, log_10_ copies per mL |  |  |
| < 5 | 37 | 1 (2.7) |
| 5-8 | 36 | 10 (27.8) |
| >8 | 2 | 0 (0.0) |
| Case presented with cough on day of exposure |  |  |
| Yes | 14 | 6 (42.9) |
| No | 61 | 5 (8.2) |

**Table S3**. Association between exposure attributes, including viral load as an ordinal variable, and a contact’s positive test result.

| Characteristic | Unadjusted RR (95% CI) | *p*-value | Adjusted RR* (95%CI) | *p*-value |
| --- | --- | --- | --- | --- |
| Index case viral load at time of test (low, medium or high)** | 1.58 (1.05 - 2.39) | 0.03 | 1.44 (1.02 - 2.04) | 0.04 |
| Time between index case test and exposure (days) | 1.12 (1.10 - 1.15) | *p* < 0.001 | 1.13 (1.11 - 1.16) | *p* < 0.001 |
| Case had cough on day of exposure | 1.85 (1.15 - 2.99) | 0.01 | 1.58 (1.15- 2.19) | *p* < 0.05 |
| Contact Type |  |  |  |  |
| Non-physical contact | Reference | - | - | - |
| Physical contact | 2.14 (1.34 - 3.44) | 0.001 | 1.95 (1.36 - 2.79) | *p* < 0.001 |

*Adjusted for viral load, time between index case test and exposure with contact, cough and contact type.

**Viral load is treated as an ordinal variable with low (<5), medium (5-8) and high values (>8) of log_10_ RNA copies per mL of saliva.

**Supplemental Methods**

Standardization curves

Standardization curves were developed using a SARS-CoV-2 standard from Exact Diagnostics (200,000 copies/mL; SKU: COV019) to prepare a series of serial dilutions resulting in 8 various copy number inputs (10,000 copies/mL; 7,500 copies/mL; 5,000 copies/mL; 2,500 copies/mL; 1,250 copies/mL; 312,5 copies/mL; 156.25 copies/mL; and 0 copies/m, respectively) in a background of pooled SARS-CoV-2-negative saliva samples. Each series of titrations was prepared in triplicate and processed in triplicate over two days, by two operators. RNA extraction was performed using the MAgMax Viral/Pathogen II Nucleic Acid Isolation kit on the automated KingFisher FlexÔ Purification System, using the MVP_saliva_Flex_200 extraction protocol (MAN0019559). Input volume sample volume was 200ul with 50ul final elution volume. RNA extraction was followed by reverse transcription and real-time PCR (RT-qPCR) using the Applied Biosystems TaqPath COVID-19 COMBO Kit with 10ul of the RNA sample. Amplification was performed using a ThermoFisher’s Applied Biosystems QuantStudio 7 Flex Real-Time PCR system equipped with Expression Suite Software v.1.3. Cycle threshold (Ct) values for each serial dilution were obtained after analysis using the Applied Biosystems’ Interpretive Software v.2. 5. Linear regression analysis was applied to derive standard curves for each of the three viral target genes of the TaqPath Assay (ORF1ab, N gene and S gene, respectively), using copy number inputs and corresponding Ct values.
